# Complexing Agent-Assisted Membraneless Zinc–Iodine Aqueous Batteries

**DOI:** 10.1021/acs.jpclett.5c01959

**Published:** 2025-07-31

**Authors:** Yutong Wu, Maxim Zhelyabovskiy, Zhitao Chen, Karam Eeso, Alexandros Filippas, Haochen Yang, Guang Yang, Nian Liu

**Affiliations:** † School of Chemical and Biomolecular Engineering, 1372 Georgia Institute of Technology, Atlanta, Georgia 30332, United States; ‡ Chemical Science Division, 6146 Oak Ridge National Laboratory, Oak Ridge, Tennessee 37831, United States

## Abstract

A membrane is required for conventional zinc–iodine aqueous batteries, since soluble polyiodides cross over to the anode side and react with zinc metal spontaneously. Making the battery membraneless increases ion transport and reduces its cost and overall footprint. In this paper, a membraneless Zn–I_2_ aqueous battery is demonstrated, employing a complexing agent, 1-butyl-1-methylpyrrolidinium iodide (MBPI), to promote the formation of I_5_^–^-containing, phase-separated polyiodides upon charging, to minimize self-discharge and suppress Zn dendrite growth. With an additional 0.3 M MBPI in 4 M ZnI_2_ electrolyte, the membraneless battery achieved 65 cycles with >85% Coulombic efficiency, whereas the MBPI-free control failed immediately. Additionally, a volumetric capacity of 14.3 Ah L^–1^ was achieved, surpassing those of most membraneless batteries reported to date regardless of redox chemistry, and underscores the potential of complexing agents in simplifying the architecture of conventional Zn–I_2_ flow batteries.

## Graphical abstract

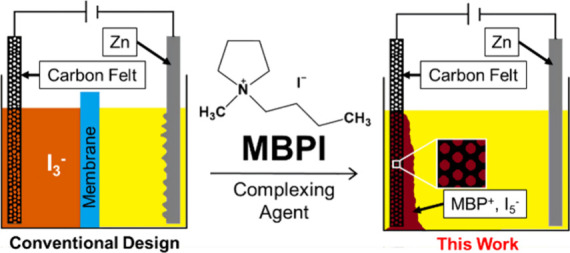

To address safety concerns associated with flammable electrolytes, aqueous-based batteries have emerged as a promising alternative, especially at the grid scale.^1–4^ Among the choices, aqueous zinc–iodine (Zn–I_2_) batteries demonstrate a high maximum theoretical energy density of up to 322 Wh L^–1^ (using a 7.0 M ZnI_2_ electrolyte). In addition, the abundance and cost of the two elements (Zn is the fourth least expensive metal that is stable in metallic form in water,^5,6^ and the concentration of I_2_ is 50 μg L^–1^ in seawater^7^) and the ambipolar nature of the electrolyte (which eliminates the need for counterions) contribute to lower capital costs, rendering Zn–I_2_ batteries a promising energy storage technology.^8,9^ Recent innovations in device architecture have even expanded the applicability of Zn–I_2_ batteries to emerging areas such as robotics and stretchable electronics.^10–14^

The high solubility of polyiodides is a double-edged sword for Zn–I_2_ batteries. On one hand, it enables a high-energy catholyte; on the other hand, it intensifies the shuttling effect of polyiodides formed during charging:

1 $$3I^{-}⇌{I_{3}}^{-}+2e^{-},E^{{}^{\circ}}=0.536\;\mathrm{V vs standard hydrogen electrode}$$

The mobile I_3_^–^ in the electrolyte can shuttle to the surface of the Zn anode and cause severe self-discharge and aggravated side reactions such as Zn morphological change and gas evolution.^15,16^ Nonporous ion-exchange membranes have been used to confine the polyiodides and tackle the self-discharge problem.^17^ Although effective, they increase cost and hinder ion transport.^18,19^ Electrode modification is utilized for tackling halogens, especially bromide crossover. However, it requires complex electrode synthesis and configuration.^20^ Using a complexing agent is another strategy to improve the performance of Zn–halogen batteries, but complexing agents forming solid products are more appropriate for solid active material-based batteries than for systems relying on liquid active materials^21,22^ and rely on complex electrode fabrication and specialized catholyte/anolyte configurations.^23^ In this Letter, we demonstrate a membraneless Zn–I_2_ battery (Figure 1) enabled by using 1-butyl-1-methylpyrrolidinium iodide (MBPI) as a complexing agent. MBPI was selected by adapting the previously established strategy of employing MEPBr in Zn–Br_2_ systems. While MEPBr has been widely used to promote the formation of higher-order polybromide species,^24^ the substitution of the ethyl group with a butyl group in MBPI was intended to enhance performance. The increased steric hindrance associated with the butyl moiety has been reported to improve complexation with both Br_2_ and I_2_ species,^25^ while the bulkier cation can also contribute to more uniform Zn deposition.^26^ MBPI promotes the formation of I_5_^–^ beyond I_3_^–^, resulting in a quasi-solid-state product that phase-separates from the aqueous electrolyte, effectively suppressing polyiodide shuttling and self-discharge. This strategy enables stable cycling at elevated state-of-charge (SOC) levels while requiring a low additive concentration, all within a simplified battery configuration that eliminates the need for complex electrode design or membrane integration for liquid active material-based batteries, such as flow batteries.

**Figure 1:**
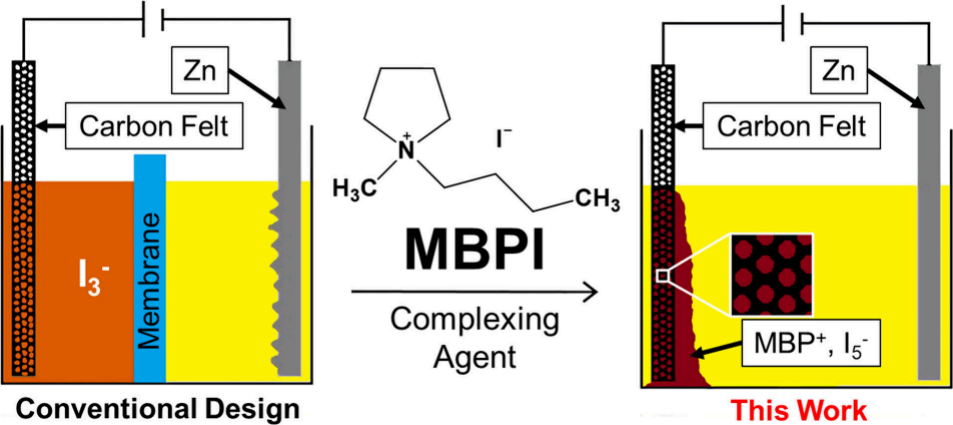
Schematic of the conventional and membraneless Zn–I_2_ battery configurations.

## Experimental Section

*Battery Assembly*. Each cathode consisted of a 5 mm × 5 mm × 70 mm piece of graphite felt (AvCarb G200) without any pretreatment or modification. For the anode, the same graphite felt was wrapped with a piece of Zn foil (Alfa Aesar). The upper sections of the electrodes were separated by a nonconductive acrylonitrile butadiene styrene plastic barrier. To prevent the electrolyte from creeping above the solution surface, a layer of wax (Apiezon W100) was applied to the graphite felt above the electrolyte level. Electrolytes were prepared by dissolving ZnI_2_ (98%, Sigma-Aldrich) to a concentration of 4 M in deionized water and adding MBPI at concentrations ranging from 0.0 to 0.3 M. The electrolyte volume was 7 mL in a 25 mm × 40 mm glass vial.

**Figure 2:**
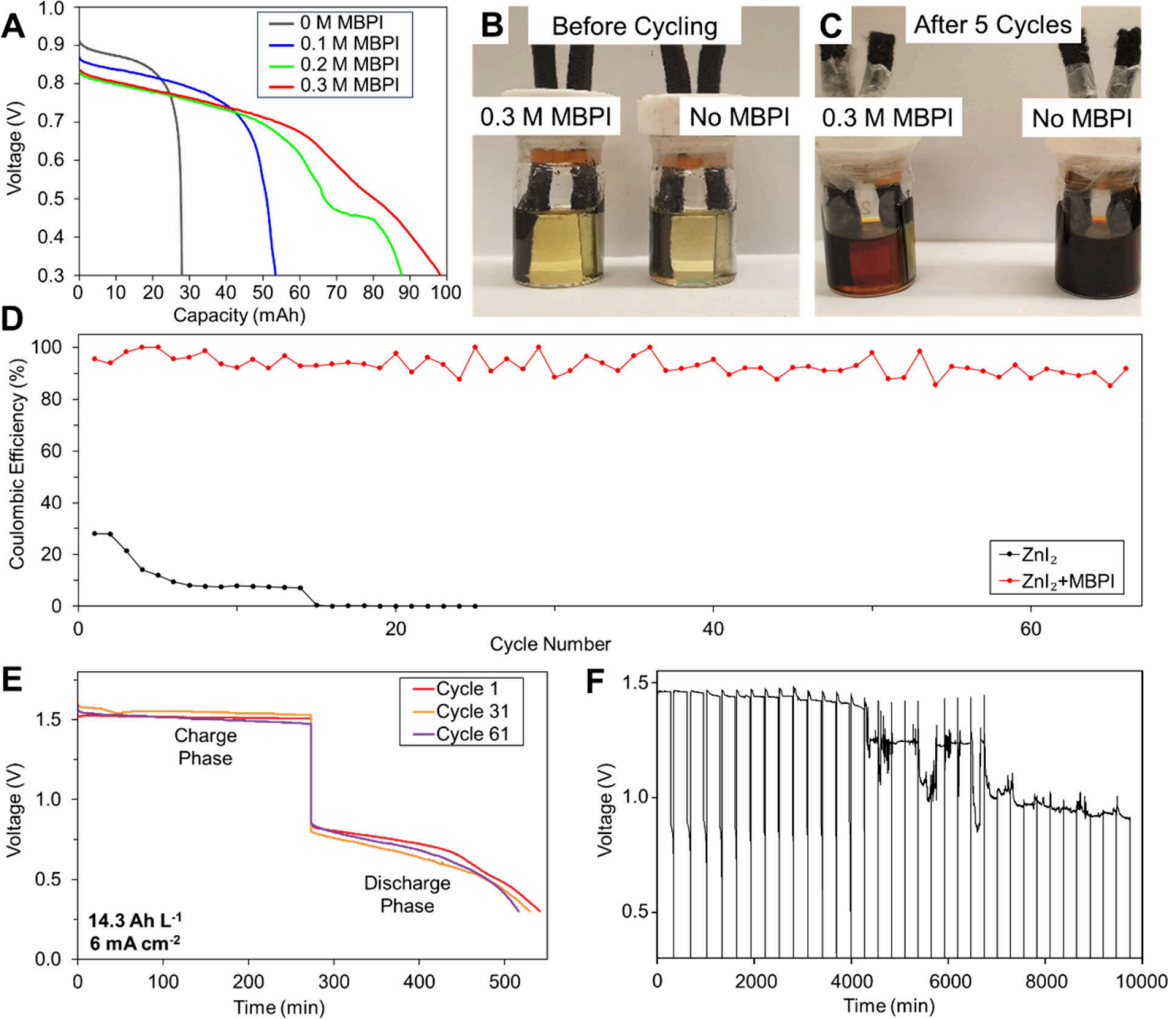
(A) Discharge capacities of cells with 4 M ZnI_2_ and 0–0.3 M MBPI electrolytes with a current density of 6 mA cm^–2^. All cells were first charged to 100 mAh (14.3 Ah L^–1^ with respect to 7 mL of electrolyte). Photos of cells with 4 M ZnI_2_/0.3 M MBPI (left) and 4 M ZnI_2_ (right) electrolytes (B) before and (C) after cycling. (D) CE of membraneless cells containing 4.0 M ZnI_2_ and 4.0 M ZnI_2_/0.3 M MBPI electrolytes. Both cells were cycled at 6 mA cm^–2^ and a constant charging capacity of 14.3 Ah L^–1^. (E) Voltage profiles of the membraneless cell containing 4.0 M ZnI_2_ and 0.3 M MBPI at different cycles. (F) Voltage profile of the 4 M ZnI_2_ cell without MBPI.

*Electrochemistry and Characterization*. Electrochemical performance was measured using a Landt CT2001A battery tester. The cells were charged to a fixed volumetric capacity of 14.3 Ah L^–1^ (SOC of 6.7%) and cycled galvanostatically with a current density of 6 mA cm^–2^ (1.26 mA cm^–3^_cathode_) unless otherwise specified. The higher cutoff voltage was 2.0 V, and the lower cutoff voltage was 0.3 V. Raman spectroscopy was conducted using a Renishaw Vis/near-IR Raman spectrometer. A 488 nm laser was used to characterize the electrolytes, while a 785 nm laser was employed to analyze the charged cathodes. Scanning electron microscopy (SEM) images of Zn anodes were acquired by using a Hitachi SU8230 instrument.

The effect of the MBPI concentration on the Coulombic efficiency (CE) of the membraneless Zn–I_2_ battery is presented in Figure 2A. Without MBPI, the Zn–I_2_ battery exhibited a CE of only 28%, indicating severe self-discharge. With the addition of 0.1, 0.2, and 0.3 M MBPI, the CE improved to 57.2%, 90.7%, and 95.5%, respectively. A concentration of 0.3 M was selected for further testing, as higher concentrations were found to degrade electrochemical performance^27,28^ and increase costs. It should be noted that the addition of MBPI reduced the concentration of free polyiodides dissolved in the electrolyte and resulted in a lower cell voltage as compared to that without MBPI. Figure 2B shows the battery setup with and without the addition of 0.3 M MBPI. No visible differences were observed, indicating that MBPI completely dissolved in the electrolyte. After prolonged cycling, the battery containing MBPI exhibited a much lighter color than the cell without MBPI, suggesting that most of the polyiodide was confined around the electrode surface rather than dissolving in the electrolyte to spontaneously react with Zn (Figure 2C).

Adding MBPI significantly improved the cycle life and CE of the Zn–I_2_ battery. Remarkably, even when cycled with a high electrolyte concentration (4 M ZnI_2_) and a high current (6 mA cm^–2^, 21 mA), the membraneless Zn–I_2_ battery with 0.3 M MBPI achieved 65 cycles (568 h in total) with >85% CE (Figure 2D). The battery containing MBPI remained stable throughout the cycling process without a membrane or separator, showing minor differences in the voltage profiles at cycles 1, 31, and 61 (Figure 2E). In comparison, the battery without MBPI cycled under the same condition with just the ZnI_2_ electrolyte was not functional at all from the beginning (<30% CE in the first cycle). The voltage profile of the battery without MBPI (Figure 2F) confirmed that the discharge capacity was negligible for each cycle until it was entirely nonfunctional due to severe self-discharge. The increased electrochemical performance of the battery with MBPI added indicated that self-discharge was effectively suppressed. Nonetheless, a small amount of free polyiodide remained in the electrolyte (as seen from the vial after cycling), contributing to mild self-discharge and Zn corrosion, which eventually led to battery failure.

To understand the complexing mechanism, Raman spectroscopy was conducted on both electrolytes before and after charging to 14.3 Ah L^–1^, as well as on cathodes after charging. The addition of 0.3 M MBPI did not affect the initial aqueous complexes before charging, indicated by the peaks representing the symmetric stretching *v*_1_(A_1_) modes of ZnI_4_^2–^ (122 cm^–1^), ZnI_3_^–^ (138 cm^–1^), and ZnI_2_ (165 cm^–1^) in panels A and B of Figure 3.^17^ No higher orders of polyiodide (I_2*n*+1_^–^, where *n* > 1) were detected. Without MBPI, a major peak at 111 cm^–1^ (I_3_^–^ symmetric stretching) appeared after charging in the electrolyte (Figure 3A), resulting in self-discharge. In contrast, the cathode charged in a 0.3 M MBPI cell showed a strong peak at 169 cm^–1^ (I–I linear stretching in I_5_^–^) and a minor peak at 111 cm^–1^ (I_3_^–^), while its electrolyte spectra continued to be dominated by Zn complexes (Figure 3B).^17,29^ These results confirm that MBPI facilitates the formation of a quasi-solid I_5_^–^ complex at the cathode, which plays a crucial role in improving the electrochemical performance of the Zn–I_2_ battery. Specifically, during the charging process, molecular I_2_ was electrochemically generated near the cathode and subsequently underwent rapid chemical complexation with I^–^ in the presence of MBPI to form the I_5_^–^ species. The resulting I_5_^–^ complexes, stabilized by the MBP^+^ cations, exhibited low solubility and precipitate out of the aqueous phase as a quasi-solid product, similar to the mechanism reported previously.^24^ This phase separation effectively isolated reactive polyiodide species from the bulk electrolyte, minimizing their diffusion toward the Zn anode and significantly suppressing self-discharge and parasitic side reactions. The confinement of polyiodides at the cathode thereby enhanced the CE and overall cycling stability.

**Figure 3:**
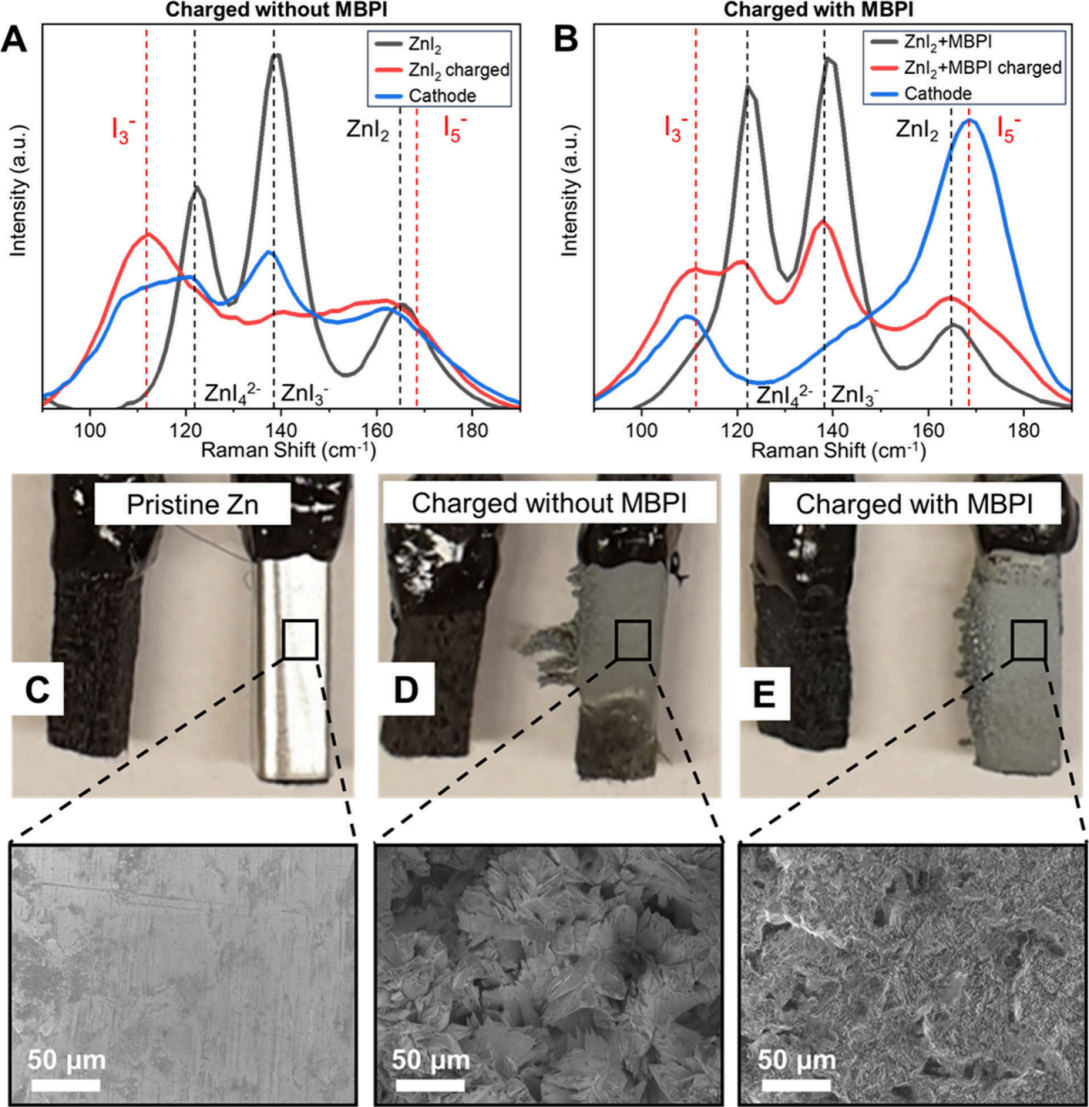
Raman spectra of the electrolytes and cathodes after charging membraneless cells containing (A) 4.0 M ZnI_2_ and (B) 4.0 M ZnI_2_/0.3 M MBPI electrolytes. (C) Photo and SEM image of the Zn anode before charging. (D) Photo and SEM image of the Zn anode after charging to 71.43 Ah L^–1^ at 6 mA cm^–2^ without MBPI. (E) Photo and SEM images of the Zn anode after charging with 0.3 M MBPI.

The introduction of MBPI into the electrolyte also has beneficial effects on the Zn anode. The pristine Zn has a smooth surface (Figure 3C); for the cell without MBPI after being deeply charged to 71.4 Ah L^–1^, visible sharp dendrites were observed and the entire bottom of the Zn anode was corroded (Figure 3D, top). In contrast, Zn dendrite formation and surface degradation were significantly suppressed in a 0.3 M MBPI cell (Figure 3E, top). The reason for this behavior is that during battery charging without MBPI, polyiodides accumulated at the bottom of the cell because of their high density and then shuttled to the Zn anode to corrode its bottom part, similar to the circumstance in the Zn–Br_2_ system.^20^ For the battery with MBPI, the polyiodides were complexed into a quasi-solid product at the cathode side and thus separated from the Zn anode, and the electrolyte contained minimal shuttling polyiodides. SEM images further revealed that the dendrites of the Zn anode charged in an electrolyte without MBPI (Figure 3D, bottom) were much larger than the Zn anode charged in the presence of MBPI (Figure 3E, bottom). This result was also due to the lower concentration of free polyiodides in the MBPI-containing electrolyte, which suppressed corrosion and preserved the fine structure.

In summary, we have demonstrated an aqueous Zn–I_2_ battery that forgos all ion-exchange and porous membranes. The complexing agent, MBPI, promoted the formation of I_5_^–^-containing products upon charging, which stayed in a quasi-solid form phase-separated from the bulk electrolyte to minimize self-discharge. The membraneless battery achieved 65 cycles with >85% CE, whereas the MBPI-free control failed immediately. Notably, our system achieved a volumetric capacity of 14.3 Ah L^–1^, which ranks among the highest reported for membraneless cells.^30^ The cationic functional groups can be tailored, such as by elongating the alkyl chain, to enhance the complexation efficacy. This strategy can be extended to other halide-based membraneless systems, depending on the specific chemistry and physicochemical properties of the storage platform (e.g., biphasic or flow systems). Combining membrane separation with halogen complexation can offer synergistic confinement of polyhalides, further suppressing crossover and enhancing electrochemical stability. Nevertheless, an optimal balance among electrochemical performance, chemical complexation, and cost remains essential.^31^
